# Tissue-Specific and Geographical Variation in Endophytic Fungi of *Ageratina adenophora* and Fungal Associations With the Environment

**DOI:** 10.3389/fmicb.2019.02919

**Published:** 2019-12-18

**Authors:** Kai Fang, Yi-Fang Miao, Lin Chen, Jie Zhou, Zhi-Ping Yang, Xing-Fan Dong, Han-Bo Zhang

**Affiliations:** ^1^State Key Laboratory for Conservation and Utilization of Bio-Resources in Yunnan, Yunnan University, Kunming, China; ^2^School of Life Sciences, Yunnan University, Kunming, China; ^3^School of Ecology and Environmental Science, Yunnan University, Kunming, China; ^4^Lu Cheng Center for Disease Control and Prevention, Changzhi, China

**Keywords:** invasive plant, endophytic fungi, tissue specific, geographic variation, air fungi, soil fungi

## Abstract

To understand the distribution of the cultivable fungal community in plant tissues and the associations of these fungi with their surrounding environments during the geographical expansion of an invasive plant, *Ageratina adenophora*, we isolated the cultivable fungi from 72 plant tissues, 12 soils, and 12 air samples collected from six areas in Yunnan Province, China. A total of 4066 isolates were investigated, including 1641 endophytic fungi, 233 withered leaf fungi, 1255 fungi from air, and 937 fungi from soil. These fungi were divided into 458 and 201 operational taxonomic units (OTUs) with unique and 97% ITS gene sequence identity, respectively. Phylogenetic analysis showed that the fungi belonged to four phyla, including Ascomycota (94.20%), Basidiomycota (2.71%), Mortierellomycota (3.03%), and Mucoromycota (0.07%). The dominant genera of cultivable endophytic fungi were *Colletotrichum* (34.61%), *Diaporthe* (17.24%), *Allophoma* (8.03%), and *Fusarium* (4.44%). *Colletotrichum* and *Diaporthe* were primarily isolated from mature leaves, *Allophoma* from stems, and *Fusarium* from roots, indicating that the enrichment of endophytic fungi is tissue-specific and fungi rarely grew systemically within *A. adenophora*. In the surrounding environment, *Alternaria* (21.46%), *Allophoma* (19.31%), *Xylaria* (18.45%), and *Didymella* (18.03%) were dominant in the withered leaves, *Cladosporium* (22.86%), *Trichoderma* (14.27%), and *Epicoccum* (9.83%) were dominant in the canopy air, and *Trichoderma* (27.27%) and *Mortierella* (20.46%) were dominant in the rhizosphere soils. Further analysis revealed that the cultivable endophytic fungi changed across geographic areas and showed a certain degree of variation in different tissues of *A. adenophora*. The cultivable fungi in mature and withered leaves fluctuated more than those in roots and stems. We also found that some cultivable endophytic fungi might undergo tissue-to-tissue migration and that the stem could be a transport tissue by which airborne fungi infect roots. Finally, we provided evidence that the fungal community within *A. adenophora* was partially shared with the contiguous environment. The data suggested a frequent interaction between fungi associated with *A. adenophora* and those in surrounding environments, reflecting a compromise driven by both functional requirements for plant growth and local environmental conditions.

## Introduction

Biological invasion has seriously threatened ecosystem services and caused huge economic losses (Vilà et al., 2010). Understanding the invasive mechanism and controlling and eradicating harmful invasive species has been a great challenge in invasive biology. In the past few decades, many hypotheses, such as the enemy release (Keane and Crawley, 2002), local pathogen accumulation (Eppinga et al., 2006), and positive plant–soil feedback (van der Putten et al., 2007, 2013) hypotheses, have proposed that soil microbes are the key factors affecting the competitiveness of exotic plants. For example, Callaway et al. (2004) reported that soil microbes were involved in negative plant–soil feedback in the growth of *Centaurea* in the plant’s native range but positive feedback outside of this range; Crocker et al. (2015) found that the pathogenic fungi *Pythium* accumulated in invaded soils and caused the death of many native plants while also indirectly enhancing the *Phragmites australis* invasion.

Relative to studies focused on rhizosphere soil fungi, few reports have characterized the endophytic fungi of invasive plants (Shipunov et al., 2008; Fischer and Rodriguez, 2013). However, all plants in nature appear to have symbioses with fungal endophytes (Rodriguez et al., 2009), and diverse fungi inhabiting plants can influence host performance, including disease resistance (Busby et al., 2016), stress tolerance (Rodriguez et al., 2008), and biomass accumulation (Mucciarelli et al., 2003). The high diversity of endophytes is indicative of their extremely complex and variable functional characteristics (Torres-Cortes et al., 2018). Endophytic fungi commonly have been categorized into two groups based on their manner of transmission: vertically transmitted endophytes (VTEs, e.g., *Neotyphodium*) that inhabit temperate grasses (*Lolium*) and are transmitted from the mother plant (Müller and Krauss, 2005) and horizontally transmitted endophytes (HTEs) transmitted mostly via spores from plant to plant (Arnold, 2007). Previous studies on native plants and crops indicated that fungal endophyte communities varied based on host genotypes (Emi and Kenji, 2013), plant tissues (Wearn et al., 2012), growth stages (Shi et al., 2016), and distribution areas (Geisen et al., 2017). However, most reports to date have addressed VTEs when exploring the endophytes of invasive plants (Rudgers et al., 2005); only very few studies have focused on the interaction between HTEs and invasive plants (Newcombe et al., 2009; Aschehoug et al., 2012). Although endophytic fungi have been proven to be a contributor to the competitiveness of exotic plants (Aschehoug et al., 2012), their variation in different tissues has not been investigated in detail during the geographical expansion of invasive plants.

In addition, it remains controversial whether endophytic fungi can grow systematically within plants. Jaber and Vidal (2010) verified that there was no systematic growth by inoculation experiments, in which the researchers could not isolate the corresponding strain from plant leaves after fungal inoculation of the roots. However, Hodgson et al. (2014) showed that systematic growth of endophytes was possible because many plant species harbored vertically transmitted fungi in seeds, and these endophytic fungi could also be recovered from the leaves. These vertically transmitted fungi have been verified to be mutualistic in promoting the invasion of temperate grasses (Rudgers et al., 2005). If horizontally transmitted endophytic fungi with positive effects can grow systematically in invasive plants, this may be an effective strategy to promote host competitiveness.

For decades, it has been demonstrated that plant species can modify the soil environment to shape rhizosphere microbes (Grayston et al., 1998), which in turn can provide feedback to promote plant growth (van der Heijden et al., 2008). Recently, some studies indicated that the phyllosphere microbes, either in living or the withered leaves, also had a strong negative feedback on plant growth (Whitaker et al., 2017), protection against pathogen damage (Ritpitakphong et al., 2016; Christian et al., 2017) and resistance to environmental stress (Saleem et al., 2017). These data highlighted the ecological importance of the microbes living in or around aboveground plant tissues. Moreover, although the enemy release hypothesis (ERH), which suggests that invasive hosts leave their enemies in their native range, partially explained some plant invasions (Keane and Crawley, 2002; Mitchell and Power, 2003), the accumulation of fungal pathogens after the introduction of exotic plants is unavoidable (Mitchell et al., 2010; Flory et al., 2013; Stricker et al., 2016). These accumulated fungal pathogens are predicted to affect neighboring native susceptible hosts if the pathogens are transmitted in the invaded ecosystem. Such dynamics are termed “spillover” when the pathogens are non-native and are introduced with the invasive plant and “spillback” when an invasive plant species hosts native pathogens (Flory et al., 2013). Previously, rare reports indicated that endophytes of invasive plants can act as local pathogens; for example, fungal endophytes, such as latent local pathogens, were isolated from the invasive plants *Parthenium hysterophorus* (Romero et al., 2001) and *P. australis* (Fischer and Rodriguez, 2013). Nonetheless, the current knowledge about the microbial community in the aboveground tissues of invasive plants as well as their association with the surrounding environment of the host is insufficient and limits the understanding of the role of fungi in plant invasion and risk evaluation.

*Ageratina adenophora* (Sprengel) R. M. King and H. Robinson is a perennial herb of the Compositae family that is native to Central America but a noxious weed in Asia, Africa, Oceania, and Hawaii. Since the first record in China in the 1940s, the plant has now become widely distributed in six provinces in Southwest China and has continuously spread east- and north-ward at a rate of approximately 20 km per year (Wang and Wang, 2006). There is evidence that strong allelopathy (Inderjit et al., 2011) and high resource capture and use efficiency (Wang et al., 2013) promote its competitive advantages over native species. Recent studies have shown that *A. adenophora* frequently changes soil microbial communities to contribute to its invasion (Xu et al., 2012; Li et al., 2017; Zhu et al., 2017). In addition, Mei et al. (2014) indicated that the diversity and isolation frequency of foliar fungal endophytes have increased with time since the introduction of *A. adenophora*.

In this study, we sampled and characterized the cultivable endophytic fungi from the roots, stems, and leaves of *A. adenophora* from six invaded areas in Yunnan Province, China, to evaluate the distribution of these fungi in different tissues in response to the geographical expansion of *A. adenophora*. We also determined whether *A. adenophora* facilitated the systematic growth of endophytic fungi. Moreover, fungi in the withered leaves and from the surrounding air and soil environments were also investigated to explore the potential interactions between the fungal communities associated with *A. adenophora* and the surrounding environments.

## Materials and Methods

### Study Site

**FIGURE 1:**
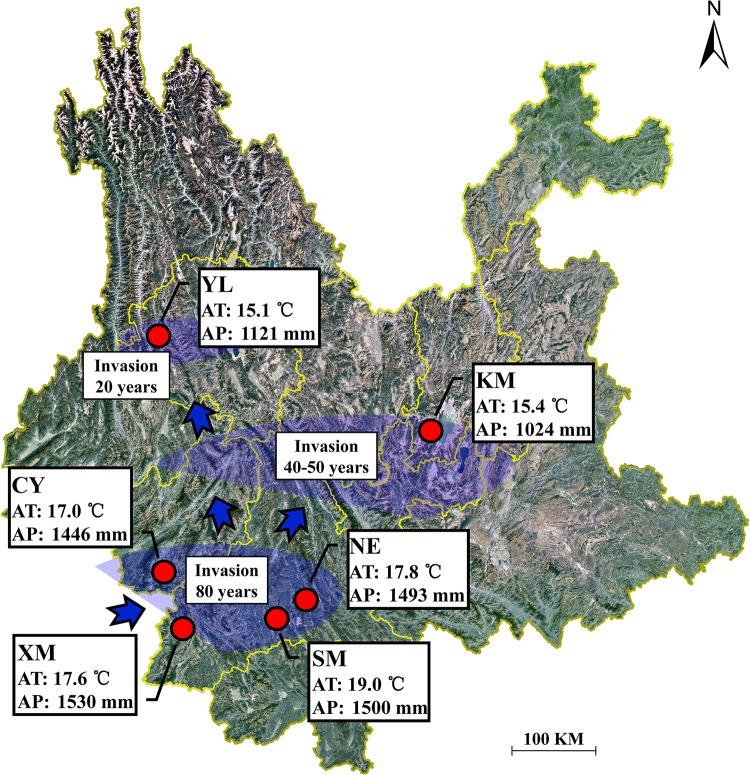
Geographical location of the sampling sites in Yunnan Province, China. AT represents the annual average temperature; AP represents the annual average precipitation.

In August 2015, during the period of the greatest vegetation growth, six *A. adenophora* populations were selected for sample collection in Yunnan Province (22.63–25.88 N, 99.31–102.37 E, 1171–2128 m), Southwest China (Supplementary Table S1). With increasing latitude, these six regions are XM, SM, NE, CY, KM, and YL, in order. The bioclimatic data were obtained from the WorldClim database using ArcGIS v10.3 (Yahdjian et al., 2011) and showed that the annual average temperature in the study area was 15.1–19.1°C and the annual average precipitation was 1024–1530 mm (Figure 1). According to the invasion dynamics of *A. adenophora* in China (Wang and Wang, 2006), the six sampling regions in this study correspond with three invasion times: >80 years ago (XM, SM, NE, and CY), ∼40–50 years ago (KM), and <20 years ago (YL) (Figure 1). In addition to *A. adenophora*, some small shrubs, herbs, and few trees occur sporadically at the sample sites (Supplementary Table S1).

### Sample Collection

**FIGURE 2:**
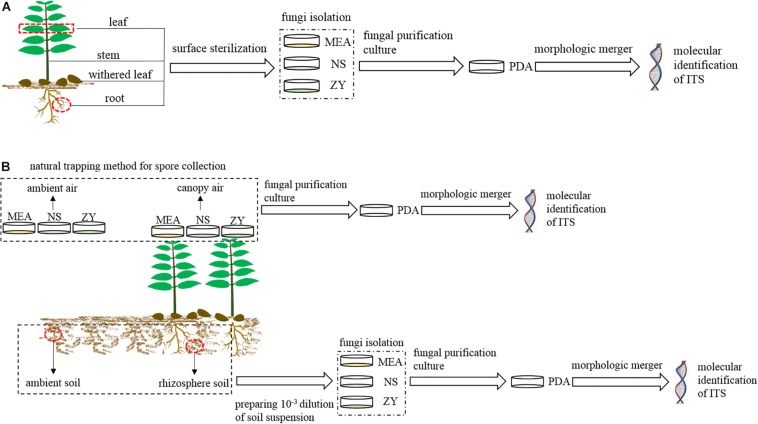
Schematic workflow of the experimental process. MEA, NS, ZY, and PDA represent the four types of culture media. **(A)** Workflow for the isolation of fungi associated with plant; **(B)**, workflow for the isolation of fungi from air and soil.

In each region, we collected four types of plant samples (roots, stems, mature, and withered leaves) (Figure 2A) and four types of environmental samples (rhizosphere and non-rhizosphere soil, canopy and non-canopy air) (Figure 2B) for fungal isolation.

For plant samples, three mature and healthy *A. adenophora* individuals were randomly selected at each region, and the fourth pair of leaves, whole stems, and fibrous roots of each plant were collected and independently packaged in sterile polypropylene bags for the isolation of endophytic fungi (Figure 2A). Moreover, six naturally withered leaves of *A. adenophora* with relatively consistent degrees of wilting were also collected and packaged in a sterile polypropylene bag for the isolation of fungi (Figure 2A). A total of 72 *A. adenophora* tissue samples were obtained from the six study areas, including 18 pairs of fully expanded leaves, and 18 stem tissue, 18 fibrous root, and 18 withered leaf samples. Fungi were isolated from all plant samples within 12 h after collection.

For environmental samples, approximately 500 g of soil was collected from the rhizosphere of *A. adenophora* and the surrounding environment in each region to isolate fungi from the rhizosphere and non-rhizosphere soil. Rhizosphere soils were collected from at least five plant individuals, and environmental soils were collected in the surrounding area approximately 10 m away from the area where *A. adenophora* was growing (Figure 2B). Both rhizosphere and environmental soils were collected at 5–15 cm underground. A total of 12 soil samples were obtained from six study areas, including six *A. adenophora* rhizosphere soil samples and six soil samples from the surrounding environment. Soil samples were taken back to the laboratory for temporary storage at 4°C, and fungal isolation was carried out within 3 days. The isolation of airborne fungi was carried out using a natural trapping method to expose the culture medium to the *A. adenophora* canopy air or ambient air for 15 min. The canopy air was considered the area with >80% coverage of *A. adenophora*, while the ambient air was considered the area with no vegetation, 10 m away from the *A. adenophora* community (Figure 2B). The fungi in the canopy and ambient air were collected approximately 60 cm above the ground. A total of 12 air samples were obtained from six study areas, including six *A. adenophora* canopy air and six ambient air samples. The Petri dishes used to collect the fungal spores from the air were sealed with Parafilm and taken back to the laboratory for subsequent pure colony culture.

### Isolation and Purification of Fungi

Both culture-dependent and culture-independent methods for fungal isolation are subject to inherent biases. For the former, the stronger competitors may emerge from tissues first, while the weaker competitors are excluded; for the latter, PCR bias may skew the representation of certain operational taxonomic units (OTUs) in the species pool (Christian et al., 2017). Although next-generation DNA sequencing has been increasingly applied to characterize fungal communities, the classification of OTUs based on sequence similarity commonly results in significant losses in taxonomic coverage (Yamamoto and Bibby, 2014), and such OTUs do not necessarily correspond to species (Ryberg, 2015). In addition, more importantly, at times fungal cultures cannot be obtained, thus stymieing the usage of these species for further ecological function experiments (Wearn et al., 2012). We thus applied culture methods in this study. Considering the preference of different fungi for different growth conditions, three types of media, namely, 2% malt extract agar (MEA) (Christian et al., 2017), NS, and ZY, were used for the cultivable fungal isolation to cover more diverse fungi. NS medium contained 1 g KH_2_PO_4_, 0.5 g MgSO_4_⋅7H_2_O, 15 g peptone, 1 g PCNB (Terraclor 75%), 0.3 g streptomycin sulfate, 0.12 g neomycin sulfate, and 20 g agar in 1000 ml distilled water. ZY medium was made as follows: 200 g of *A. adenophora* healthy leaves was collected from the wild and cleaned and then boiled in 1000 ml deionized water for approximately 30 min; 15 g sucrose and 18 g agar were then added to the collected filtrate.

Endophytic fungi (roots, stems, and mature leaves) and fungi from withered leaves were isolated by placing surface sterilized tissue fragments on the culture medium (Figure 2A). First, plant samples were rinsed with tap water and then surface sterilized as follows: leaves (including mature and fallen) were submerged in 70% ethanol for 2 min, 0.5% sodium hypochlorite for 2 min, and rinsed with sterile water six times; stems were submerged in 70% ethanol for 2 min, 0.5% sodium hypochlorite for 5 min, 70% ethanol for 1 min, and rinsed with sterile water six times; roots were submerged in 70% ethanol for 5 min, 0.5% sodium hypochlorite for 5 min, and rinsed with sterile water six times. The sterile water used for the last rinse was inoculated into potato dextrose agar (PDA, 1000 ml distilled water containing 200 g potato, 20 g dextrose, and 20 g agar) medium to verify the surface sterilization. Subsequently, the same tissues collected at the same site were mixed and cut into pieces, with leaves and stems approximately 6 mm^2^ in size and roots approximately 0.2 cm in length. Sixteen pieces from each mixed sample were haphazardly selected and placed in a 10-cm-diameter Petri plate containing either 2% MEA, NS, or ZY medium. Soil fungi were isolated by preparing soil suspensions at a 10^–3^ dilution and uniformly plating the suspensions on 2% MEA, NS, or ZY medium (Figure 2B). Airborne fungi were isolated from the Petri dishes that collected airborne spores by the natural trapping method in the field (Figure 2B).

The culture procedures were repeated three times for each medium of the same sample, and 216 Petri plates (162 for endophytic fungi and 54 for withered leaf fungi) were used to isolate fungi from *A. adenophora* tissues, 108 Petri plates were used to isolate soil fungi, and 108 Petri plates were used to isolate air fungi. The Petri plates were sealed with Parafilm and incubated in a growth chamber in dark conditions at a constant temperature of 28°C. Plates were monitored daily and the occurrence of fungal colonies was recorded as they emerged. The colonies on each plate were counted and classified into morphotypes as described previously (Glynou et al., 2016), and the representative strain of each morphotype was transferred to a new plate containing PDA for pure cultivation and for further colony morphological classification. In total, we counted 4066 colonies from three types of media and classified them into 1072 groups (Supplementary Table S2). Then, these representative fungal strains were subjected to molecular identification of ITS sequences. All representative strains were stored in the laboratory of Yunnan University for future experiments.

### Molecular Identification of Fungi

Total genomic DNA was extracted from fungal mycelia using the CTAB method (Stewart and Via, 1993). The primers ITS4 and ITS5 were used to amplify the ITS region of the fungal DNA. Each 50 μl PCR included 5 μl 10× amplification buffer, 5 μl dNTP mixture, 1 μl each primer (10 μM), 0.25 μl Taq DNA polymerase, 1 μl template DNA, and 37 μl ddH_2_O. The amplification was run in a Veriti^®^ 96-Well Thermal Cycler (Applied Biosystems Inc., Foster City, CA, United States) [4 min at 94°C, followed by 35 cycles (1 min at 94°C, 1 min at 54°C, and 1 min at 72°C), 10 min at 72°C]. PCR products were tested by 1% gel electrophoresis and then sent to the Beijing Genomics Institute (BGI) for ITS sequencing.

Based on the GenBank database, sequence homology analysis, quality assessment, and correction were conducted. ClustalX 2.1 (Thompson et al., 1997) was used to cut out chimeric bases to make each sequence approximately 550 bp in length. The Mothur program was used to identify the remaining sequences and group the consensus sequences into OTUs at unique and 97% sequence identity (Schloss et al., 2009). The nucleotide sequences reported in this study have been deposited in GenBank under the accession numbers MK303973–MK304430.

### Statistical Analyses

All statistical analyses were performed at a level of 100% (unique) and 97% ITS sequence identity. We pooled the colonies from the three plates of each medium, and the three types of medium were used as biological replicates for each sample because there were no significant differences in the community composition (Supplementary Figure S1), species diversity (Supplementary Figures S2A,B), and isolate abundance (Supplementary Figure S2C) among the three types of medium, with the exception that fungal community variation was significantly associated with the medium at 97% sequence identity (Supplementary Figure S1B; *F* = 2.2, *p* = 0.002) but only explained very little variation [0.8% adjusted explained variation (AEV)]. Communities were defined as the sequenced fungal strains obtained from a given plant tissue, site, or environmental source.

Non-metric multidimensional scaling (NMDS) was used to visualize the similarity of fungal communities among plant tissues or environmental samples, and principal coordinate analysis (PCoA) was used to assess the spatial variation of endophytic and withered leaf fungal communities in different plant tissues during the geographical expansion of *A. adenophora*. At the unique sequence identity level, two outliers [NE–RS–NS and NE–AS–MEA; NE represents the sampling site, RS (rhizosphere soil) and AS (ambient soil) represent the source, and NS and MEA represent the culture medium; detailed in Supplementary Table S2] were eliminated in the NMDS analysis of the environmental samples. The correlation between fungal communities and the classified factors was tested by redundancy analysis (RDA). All distance matrices for community composition analyses were based on the Bray–Curtis dissimilarity index.

The Shannon diversity index was calculated for fungi from both plant tissue and the environment. ANOVA was used to compare the diversity and abundance of fungi (containing endophytic and withered leaf fungi) across plant tissues and geographical sites. Assumptions of normality were tested by Kolmogorov–Smirnov test, and the assumptions were met. *Post hoc* comparisons were performed using Duncan’s tests for equal variance and Dunnett’s T3 tests for unequal variance. Linear regression was used to analyze the relationship between the Shannon diversity index or average abundance and latitude. Mann–Whitney *U* non-parametric tests were used to compare the diversity and average abundance of fungi from different environmental sources. Fungal relative abundance was calculated to analyze the relationship between plant tissue and environmental fungi.

Multivariate analyses were performed using CANOCO, version 5.0 (Ter Braak and Šmilauer, 2012), and all other analyses were executed using SPSS version 22.0 (SPSS Inc., Chicago, IL, United States). Visualization of diversity and abundance data were realized with GraphPad Prism 7 (GraphPad Software Inc., San Diego, CA, United States).

## Results

### Phylogenetic Analysis

A total of 4066 cultivable isolates were counted and investigated, including 1641 endophytic fungi, 233 withered leaf fungi, 1255 air fungi, and 937 soil fungi. Based on their colony morphologies, these fungi were first classified into 1072 groups, and the representative strains from each group were sequenced into the ITS gene and then divided into 458 and 201 OTUs at unique and 97% sequence identity, respectively (Supplementary Table S3). These isolates were placed into four phyla, including Ascomycota (94.20%), Basidiomycota (2.71%), Mortierellomycota (3.03%), and Mucoromycota (0.07%). The isolates in Ascomycota were further divided into five classes, including Dothideomycetes (34.07%), Eurotiomycetes (5.93%), Leotiomycetes (0.47%), Pezizomycetes (0.13%), and Sordariomycetes (59.37%). The dominant genera of cultivable endophytic fungi were *Colletotrichum* (34.61%), *Diaporthe* (17.24%), *Allophoma* (8.03%), and *Fusarium* (4.44%). *Colletotrichum* and *Diaporthe* were primarily isolated from mature leaves, *Allophoma* from stems, and *Fusarium* from roots. The dominant genera differed in the withered leaves and were represented by *Alternaria* (21.46%), *Allophoma* (19.31%), *Xylaria* (18.45%), and *Didymella* (18.03%). In addition, *Cladosporium* (22.86%), *Trichoderma* (14.27%), and *Epicoccum* (9.83%) were the most abundant cultivable fungi in the canopy air, but *Trichoderma* (27.27%) and *Mortierella* (20.46%) were dominant in the rhizosphere soil.

### Tissue Specificity in Cultivable Fungal Communities

**FIGURE 3:**
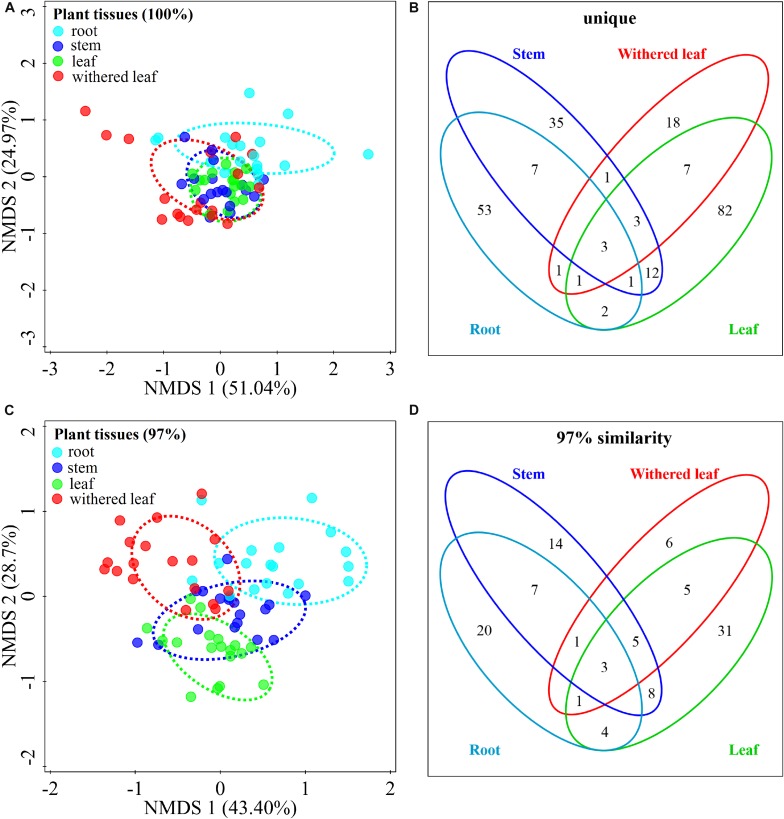
Tissue specificity of cultivable fungal communities in *A. adenophora*. Non-metric multidimensional scaling (NMDS) analysis shows the similarity among cultivable fungal communities (points) [**A** (unique), stress = 0.083; **C** (97%), stress = 0.112], and the Venn diagram shows the amount of the same or different OTUs among plant tissues [**B** (unique), **D** (97%)]. Percentages of total variation explained by the NMDS axes are given in parentheses.

**FIGURE 4:**
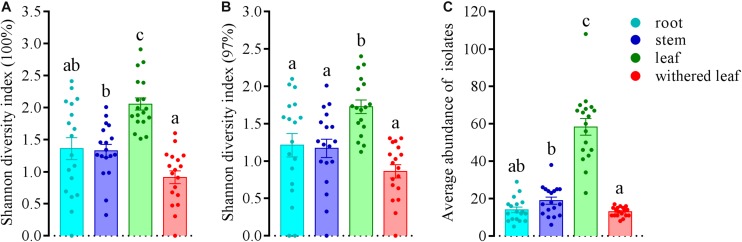
Variation in Shannon diversity index values at unique **(A)** and 97% sequence identity levels **(B)** and average abundance **(C)** in cultivable fungal communities among different tissues of *A. adenophora*. The Kolmogorov–Smirnov normality test was satisfied. Dunnett’s T3 test was used for multiple comparisons. Different lowercase letters indicate significant differences (*p* < 0.05). Error bars depict the standard errors.

The community composition (Figures 3A,C) of cultivable endophytic fungi in *A. adenophora* showed tissue specificity, while there was systematic growth of a few strains in plants (Figures 3B,D). However, there was no consistent trend for the OTUs at unique and 97% sequence identity. Although there was a significant correlation between plant tissue and fungal community variation both at the unique [*F* = 2.8; *p* = 0.002; 3.8% explained variation (EV), 2.5% AEV] and 97% (*F* = 5.1; *p* = 0.002; 6.7% EV, 5.4% AEV) sequence identity levels, community variations between different tissues were more distinct at the 97% level (Figure 3C), while the unique sequences only showed such variation between above- and belowground tissues (Figure 3A). The closeness of plant tissues appears to impact the composition of the fungal community, i.e., the endophytic community composition between roots, stems, and leaves was more similar (Figures 3A,C), and the number of identical fungi between stems and roots was higher than that between leaves and roots (Figures 3B,D). In addition, Shannon diversity (Figures 4A,B) and average abundance (Figure 4C) showed variation among different tissues, among which mature leaves had the highest levels, while the withered leaves had the lowest levels.

### Geographical Variation in Cultivable Fungal Communities

**FIGURE 5:**
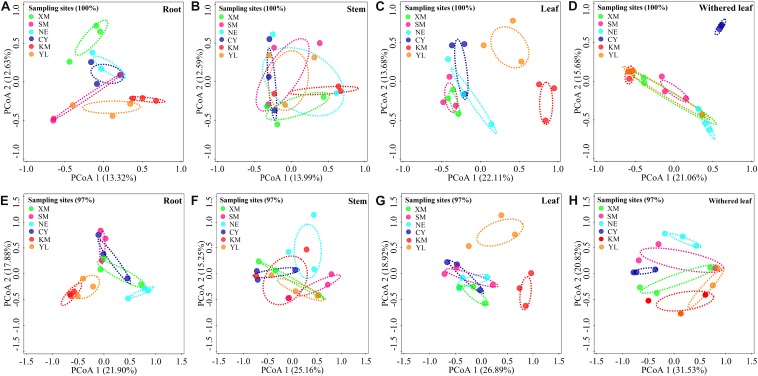
Geographical variation in cultivable fungal communities in roots [**A** (unique), **E** (97%)], stems [**B** (unique), **F** (97%)], leaves [**C** (unique), **G** (97%)], and withered leaves [**D** (unique), **H** (97%)] of *A. adenophora*. Principal co-ordinate analysis (PCoA) depicts the similarity among cultivable endophytic fungal communities (points). Percentages of total variation explained by the PCoA axes are given in parentheses.

**FIGURE 6:**
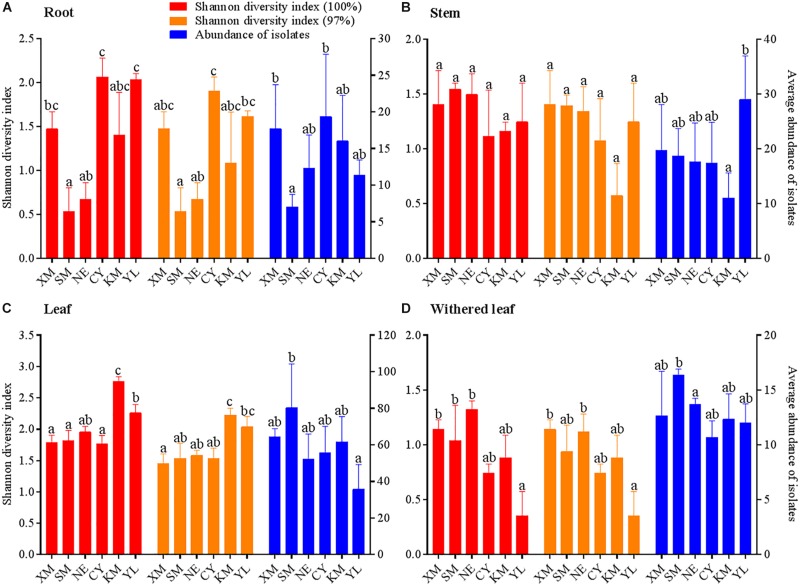
Geographical variation in Shannon diversity index values and average abundance of cultivable fungal communities in different tissues (**A**, roots; **B**, stems; **C**, leaves; **D**, withered leaves) of *A. adenophora*. The Kolmogorov–Smirnov normality test was satisfied. Dunnett’s T3 test was used to compare the geographical variation in the average fungal abundance of the withered leaves, and Duncan’s test was used for all other multiple comparisons. Different lowercase letters indicate significant differences (*p* < 0.05). Error bars depict the standard errors.

Cultivable fungal communities in plant tissues varied among geographic sites (Figures 5, 6) at either the unique or 97% sequence identity level. The six sampling sites could be divided into three groups of low (including XM, SM, NE, and CY, latitude from 22.63 to 23.22), middle (KM, latitude 24.54), and high (YL, latitude 25.88) latitudes. RDA showed that the latitude was an important factor for explaining the differences in composition in cultivable fungal communities among roots (Figure 5A: *F* = 1.7; *p* = 0.006; 9.8% EV, 4.1% AEV; Figure 5E: *F* = 2.9; *p* = 0.002; 15.4% EV, 10.1% AEV), leaves (Figure 5C: *F* = 3.0; *p* = 0.002; 16.0% EV, 10.7% AEV; Figure 5G: *F* = 3.6; *p* = 0.002; 18.3% EV, 13.2% AEV), and withered leaves (Figure 5D: *F* = 1.7; *p* = 0.044; 9.5% EV, 3.9% AEV; Figure 5H: *F* = 2.8; *p* = 0.006; 14.7% EV, 9.4% AEV) but not among stems (Figure 5B: *F* = 1.3; *p* = 0.118; 7.5% EV, 1.7% AEV; Figure 5F: *F* = 1.4; *p* = 0.172; 8.2% EV, 2.5% AEV).

**FIGURE 7:**
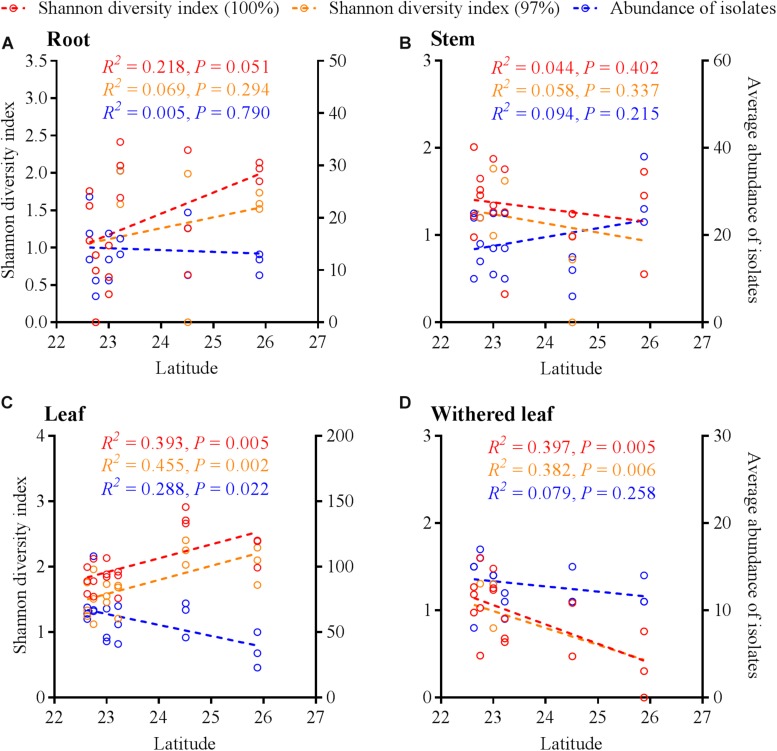
Linear regression of latitudes with Shannon diversity index values and average abundance of cultivable fungi in *A. adenophora* different tissues (**A**, roots; **B**, stems; **C**, leaves; **D**, withered leaves).

Similar to the community composition, except for the cultivable endophytic fungi of the stems (Figure 6B), the Shannon diversity index and average abundance in the other three plant tissues were significantly different among the sampling sites (Figures 6A,C,D), but the same linear relationship with latitude was not observed (Figure 7). For example, the latitude positively correlated with the diversity of the leaf fungi (Figure 7C) but negatively correlated with the diversity of the withered leaf fungi (Figure 7D) and with the abundance of the leaf fungi (Figure 7C).

### Association of Cultivable Fungal Communities With the Surrounding Environments

**FIGURE 8:**
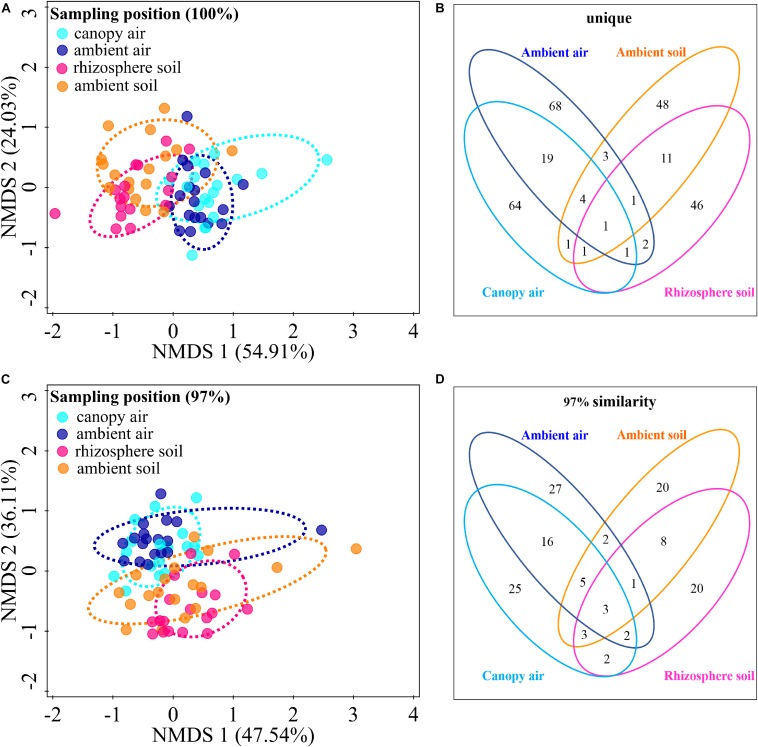
Effects of *A. adenophora* invasion on the cultivable fungal communities in air and soil. Non-metric multidimensional scaling (NMDS) analysis shows the similarity among cultivable fungal communities (points) [**A** (unique), stress = 0.052; **C** (97%), stress = 0.102], and the Venn diagram shows the amount of the same or different OTUs among environment samples [**B** (unique), **D** (97%)]. There were two outlier samples [NE–RS–NS and NE–AS–MEA; NE represents the sampling site, RS (rhizosphere soil) and AS (ambient soil) represent the source, and NS and MEA represent the culture medium. See Supplementary Table S2 for details]. at the unique sequence identity level **(A)**. To better visualize the primary data, the NMDS analysis shown in panel **A** does not take these two outliers into account. Percentages of total variation explained by the NMDS axes are given in parentheses.

**FIGURE 9:**
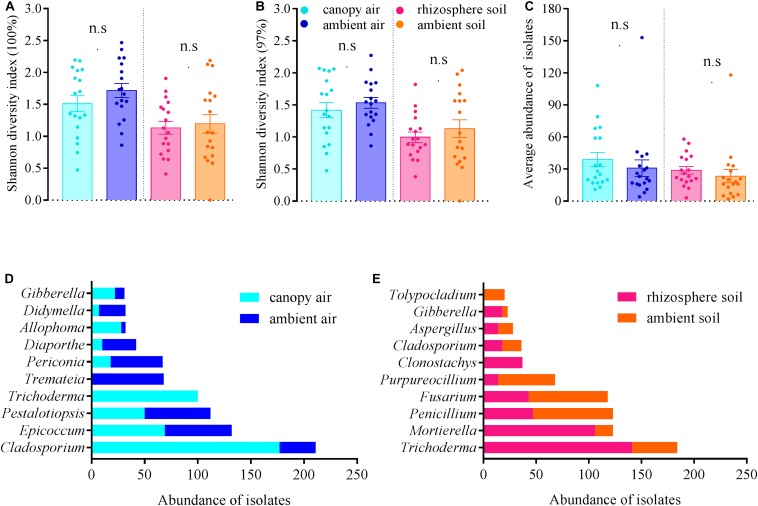
Effects of *A. adenophora* on Shannon diversity index values [**A** (unique), **B** (97%)], community average abundance **(C)**, and total abundance of dominant cultivable fungal genera **(D,E)** in air and soil. Non-parametric Mann–Whitney *U*-test was used for the test of two independent samples (canopy air vs. ambient air, rhizosphere soil vs. ambient soil), and n.s. indicates no significant difference (*p* > 0.05). Error bars depict the standard errors.

The cultivable fungal community composition was significantly different between air and soil at both unique (Figure 8A, *F* = 4.1; *p* = 0.002; 5.7% EV, 4.3% AEV) and 97% (Figure 8C, *F* = 7.4; *p* = 0.002; 9.6% EV, 8.3% AEV) sequence identity levels, with a few shared strains in both environments (Figures 8B,D). However, *A. adenophora* invasion only marginally changed the composition (Figures 8A,C), Shannon diversity index (Figures 9A,B), and average abundance (Figure 9C) of cultivable fungal communities, either in canopy air or rhizosphere soils. RDA showed that the cultivable fungal community was only significantly different between the rhizosphere and the ambient soils at 97% sequence identity (*F* = 1.7; *p* = 0.034; 4.7% EV, 1.8% AEV), while other cases were not significantly different [air (unique): *F* = 1.1; *p* = 0.282; 3.0% EV, 0.2% AEV; air (97%): *F* = 0.9; *p* = 0.612; 2.5% EV, 0.0% AEV; soil (unique): *F* = 1.4; *p* = 0.07; 4.1% EV, 1.1% AEV]. Nonetheless, *A. adenophora* caused certain fungal fluctuations in surrounding environments at the genus level (Figures 9D,E); for example, *Cladosporium*, *Trichoderma*, *Allophoma*, and *Gibberella* were much more abundant in canopy than in ambient air (Figure 9D), and *Trichoderma* and *Mortierella* were three and six times higher in rhizosphere soil than in ambient soil (Figure 9E).

**FIGURE 10:**
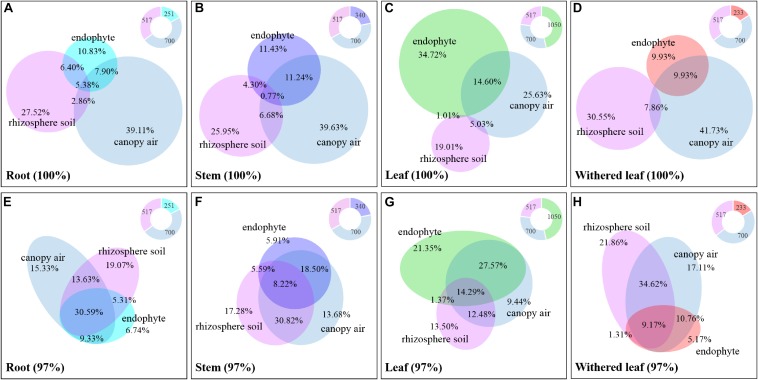
Relative abundance of the cultivable fungi among plant tissues, canopy air, and rhizosphere soil. Panels **A** (unique) and **E** (97%) represent roots, panels **B** (unique) and **F** (97%) represent stems, panels **C** (unique) and **G** (97%) represent leaves, and panels **D** (unique) and **H** (97%) represent withered leaves. The overlapping parts of solid circles or ellipses with different colors represent the same OTUs, and the percentage is the relative abundance. The different colored parts of the ring diagrams show the abundance of cultivable endophytic fungi, rhizosphere fungi, and canopy air fungi, respectively.

The cultivable fungi in aboveground tissues (stems, leaves, and whithered leaves) were closely related to those in contiguous environments, especially at 97% sequence identity (Figure 10 and Supplementary Figure S3). For example, foliar endophytic fungi shared 14.60% OTUs with canopy air but only 1.01% with rhizosphere soils at a unique level (Figure 10C), and these two percentages increased to 41.86 and 15.66%, respectively, at 97% sequence identity (Figure 10G). Similarly, the withered leaves shared 9.93% of cultivable fungi with canopy air but no sharing occurred with rhizosphere soil fungi at the unique identity level (Figure 10D). Interestingly, both roots and stems shared larger proportion of fungi with canopy air than with rhizosphere soils (Figures 10A,B,E,F). In total, the proportion of shared fungi was larger when comparing plant tissues than when comparing plant tissue with environments, and when comparing plant tissues with canopy air than comparing plant tissues with soils (Supplementary Figure S3). A few fungi in the aboveground tissues, which were not isolated from the contagious canopy air, were shared with soils (Figure 10 and Supplementary Figure S3).

## Discussion

The horizontally transmitted endophytic fungi usually differ among plant species in a certain habitat (Emi and Kenji, 2013) and vary among different tissues within an individual plant (Wearn et al., 2012). In this study, the cultivable endophytic fungal community of *A. adenophora* showed obvious tissue specificity (Figures 3, 4), consistent with most previous results (Wearn et al., 2012; Yan et al., 2015; Geisen et al., 2017). However, our study further detailed that the cultivable endophytic fungi, as well as the withered leaf cultivable fungi, changed with the geographic expansion of *A. adenophora* and showed a certain degree of variation in different tissues (Figures 5, 6). Relative to the roots and stems, the mature and withered leaves showed more significant fluctuations of the cultivable fungi with geographic latitude (Figure 7). In a given tissue, the change pattern was also different for the investigated fungal parameter. For example, the fungal diversity was positively correlated but the abundance was negatively correlated with latitude (Figure 7C). These change patterns may be related to the selectivity of functional requirements in the assembly of endophytic fungi in plant tissues (Torres-Cortes et al., 2018) under different geographic backgrounds (Martiny et al., 2006; Rodriguez et al., 2009; Yokoya et al., 2017). In general, fungal endophyte communities vary across latitudinal gradients (Arnold and Lutzoni, 2007). The endophytic fungi of exotic plants, even with vertical transmission, also change with the expansion of exotic plants to high latitudes (Geisen et al., 2017). It is very complex to decipher the factors influencing the fungal change pattern along with the northward invasion of *A. adenophora*; however, more fluctuations were observed in fungi inhabiting leaf tissues than those inhabiting roots and stems (Figure 7), suggesting that leaves may face strong fluctuations in environmental temperature stress during *A. adenophora* expansion (Figure 1). Similarly, Kazenel et al. (2019) also indicated that leaf fungi varied more strongly with altitude or warming than root fungi, suggesting that leaf symbioses may be particularly susceptible to disruption under climate change, perhaps because they are less buffered against air temperatures than belowground fungi.

Lodge et al. (1996) and Rodriguez et al. (2009) described fungal endophyte species as a mosaic of infections in a leaf, implying that plant infection with endophytes was limited by the random dynamics of microbial resources in the environment as well as the selective effects of plant species or plant tissues. Because Yan et al. (2015) demonstrated that there was very little evidence for any systematic growth of foliar fungal endophytes within an individual plant, and the main driving process behind the assembly of foliar fungal endophytes was random. We found that the cultivable fungi in plant tissues were shared with those from both canopy air and rhizosphere soil, representing the important role of random environmental dynamics in the composition of cultivable fungal communities in *A. adenophora*. This role was also supported by the greater number of fluctuations in fungi inhabiting leaf tissues than in those inhabiting roots and stems (Figure 7). Moreover, there was a higher percentage of shared fungi in aboveground tissues and canopy air than in roots and rhizosphere soils (Figure 10 and Supplementary Figure S3), indicating that the environmental inoculation sources played a more important role in the fungal assembly of aboveground parts than in that of the belowground parts. Interestingly, even the assemblage of fungal communities in plant tissues may be a compromise driven by both functional requirements for plant growth (Torres-Cortes et al., 2018) and local environmental conditions, e.g., the temperature (Campisano et al., 2017); the former may weight out the latter because we found that the fungal similarity within tissues was much higher than that between tissues on a relatively large geographical scale (Figure 3).

Within a host, some studies have demonstrated endophytic bacterial migration through tissues (Chi et al., 2005; Tharek et al., 2011). For example, Martí et al. (1999) demonstrated that *Agrobacterium tumefaciens* was recovered from both roots and crowns 3 months after inoculation of rose plant stems. There has been no report about horizontally transmitted fungal endophyte migration in exotic plants. We found a large number of shared fungi between rhizosphere soil, plant tissue, and canopy air (Figure 10 and Supplementary Figure S3); the cultivable fungal community composition was related to the tissue connection; that is, both the similarity and the number of the shared fungi between roots and stems were higher than those between roots and leaves (Figure 3 and Supplementary Figure S3). Again, the portion of shared root endophytic fungi was explained not only by those from the rhizosphere soil but also by a high proportion of those from air fungi (Figures 10A,E and Supplementary Figure S3). We also isolated a small proportion of soil fungi in the mature leaves (Figures 10C,G and Supplementary Figure S3). These facts indirectly indicated that some endophytic fungi of *A. adenophora* might undergo tissue-to-tissue migration and that the stem could be a transport tissue for fungal travel from the air to leaves and then to the roots or *vice versa.* These findings thus introduce an interesting topic, i.e., the study of the transport of fungi in stems for understanding the interaction of endophytic fungi with the host as well as the surrounding environment including the soil and canopy air.

Multiple studies have shown significant impacts of invasive plant populations on soil microbial communities (Xu et al., 2012; Li et al., 2017; Malinich et al., 2017); however, such an effect on airborne microbes has received no attention. In fact, plants contribute microbes to the air around them (Lymperopoulou et al., 2016). In this study, there was no significant effect of *A. adenophora* on fungi at the community level, both in rhizosphere soil and canopy air (Figures 8A,C, 9A–C); however, *A. adenophora* changed the dominant genera (Supplementary Table S3) and the total abundance of certain strains (Figures 9D,E). The data indicated that certain fungal taxa, e.g., *Cladosporium*, *Trichoderma*, *Allophoma*, and *Gibberella*, may have been released into the air from the leaf surface by *A. adenophora*. In recent years, studies on the interaction between the phyllosphere microbiome and plants have emphasized the importance of microbes inhabiting aboveground tissues in ecology (Ritpitakphong et al., 2016; Christian et al., 2017; Saleem et al., 2017; Whitaker et al., 2017). Therefore, it is both important and interesting to explore the potential ecological effects of certain fungi released into the air by *A. adenophora*, e.g., these fungi, if they are local pathogens, may increase the risk of disease infection in neighboring plants (also see the section “Discussion”).

Currently, the interactions between endophytic fungi and their hosts have been considered diverse, and evaluating their ecological consequences is complex (Newsham, 2011). Interestingly, it has been recently reported that many fungi, such as *Colletotrichum*, *Fusarium*, and *Verticillium*, can not only inhabit some plant species as endophytes but also have pathogenic effects on other plant species (Arnold, 2007). For example, *Colletotrichum magna*, a pathogen of Cucurbitaceae plants, often exists in other non-Cucurbitaceae plants as an endophyte (Kogel et al., 2006). Many crop pathogens also frequently lurk in other weeds in an endogenous form (Photita et al., 2004). Previously, fungal endophytes, as latent local pathogens, were isolated from the invasive plants *P. hysterophorus* (Romero et al., 2001) and *P. australis* (Fischer and Rodriguez, 2013). The possible enrichment of endophytic fungi that may act as local pathogens by invasive plants can undoubtedly serve as a weapon to indirectly promote plant invasion. The most abundant endophytic fungi isolated from *A. adenophora* were *Colletotrichum* (Supplementary Table S1), many of which have been reported as generalist pathogens (O’Connell et al., 2012), accounting for >50% of the foliar endophytic fungi. Therefore, it is worth evaluating whether the direct and indirect effects of the spillback of accumulated fungi on neighboring plants finally become a competitive advantage for *A. adenophora*. In addition, compared with the fungi in ambient air and soil, more abundant *Trichoderma* were isolated in the rhizosphere soil and canopy air of *A. adenophora* (Supplementary Table S3 and Figures 9D,E). Since at least the 1920s, *Trichoderma* species have been known for their ability to act as biocontrol agents against plant pathogens (Harman, 2006). It also remains to be explored whether the resistance of these *Trichoderma* to pathogens indirectly improves the competitiveness of *A. adenophora*.

Methodologically, because inherent biases are unavoidable for both the culture-dependent and culture-independent methods of investigating fungal diversity (Christian et al., 2017), we selected culture methods primarily to be able to use these cultivable fungi to test their ecological functions in *A. adenophora* invasion in later experiments. Actually, our small-scale investigation of *A. adenophora* fungi not only revealed that the fungal communities were indeed different between the culture-dependent and culture-independent methods but also that there was a significant tissue specificity (Zhou et al., 2019). Therefore, it is still necessary to combine these two methods for deciphering fungal distribution and interaction inside and outside of *A. adenophora.* In addition, OTU classification has been widely used in fungal community studies based on the ITS sequence similarity at 100 (Geisen et al., 2017), 99 (Sarmiento et al., 2017), and 97% (Christian et al., 2017), and this usually results in distinct fungal diversity estimates as well as ecological hypotheses that arise from such observations (Gazis et al., 2011). Similarly, in this study, 100% sequence identity only showed an above- and belowground differentiation in fungal community composition (Figure 3A), while use of the 97% sequence identity showed the complete differentiation among roots, stems, leaves, and withered leaves (Figure 3C). Although the comparison of the two OTU classification criteria also showed a slight impact for the Shannon diversity index values (Figures 4, 6), the primary conclusions of this study were not affected.

## Conclusion

In conclusion, we found that the invasive plant *A. adenophora* enriched the cultivable endophytic fungi, which showed a significant tissue specificity, and demonstrated that the fungi rarely occurred systemic growth. Our study further revealed that the cultivable endophytic fungi of *A. adenophora* changed across geographic areas but showed a certain degree of variation in different tissues. Relatively, the cultivable fungi in mature and withered leaves showed more fluctuations than those in roots and stems. We also found that some cultivable endophytic fungi of *A. adenophora* might undergo tissue-to-tissue migration, and the stem could be a transport tissue by which airborne fungi infect roots or *vice versa*. Finally, we found that the composition of the cultivable fungal community was related to the contiguous environment, which reflects a frequent interaction between fungi associated with *A. adenophora* and those in surrounding environments.

## Data Availability Statement

The datasets generated for this study can be found in GenBank, MK303973–MK304430.

## Author Contributions

KF analyzed the data and wrote the manuscript. Y-FM designed the project, analyzed the data and performed the experiments. KF, LC, JZ, Z-PY, and X-FD collected the samples and performed the experiments. H-BZ designed the project and wrote the manuscript. All authors have read and approved the submission of this manuscript.

## Conflict of Interest

The authors declare that the research was conducted in the absence of any commercial or financial relationships that could be construed as a potential conflict of interest.

## References

[B1] Arnold A. E. (2007). Understanding the diversity of foliar endophytic fungi: progress, challenges, and frontiers. *Fungal Biol. Rev.* 21 51–66. 10.1016/j.fbr.2007.05.003

[B2] Arnold A. E. Lutzoni F. (2007). Diversity and host range of foliar fungal endophytes: are tropical leaves biodiversity hotspots? *Ecology* 88 541–549. 10.1890/05-1459 17503580

[B3] Aschehoug E. T. Metlen K. L. Callaway R. M. Newcombe G. (2012). Fungal endophytes directly increase the competitive effects of an invasive forb. *Ecology* 93 3–8. 10.1890/11-1347.1 22486080

[B4] Busby P. E. Ridout M. Newcombe G. (2016). Fungal endophytes: modifiers of plant disease. *Plant Mol. Biol.* 90 645–655. 10.1007/s11103-015-0412-0 26646287

[B5] Callaway R. M. Thelen G. C. Rodriguez A. Holben W. E. (2004). Soil biota and exotic plant invasion. *Nature* 427 731–733. 10.1038/nature02322 14973484

[B6] Campisano A. Albanese D. Yousaf S. Pancher M. Donati C. Pertot I. (2017). Temperature drives the assembly of endophytic communities’ seasonal succession. *Environ. Microbiol.* 19 3353–3364. 10.1111/1462-2920.13843 28654220

[B7] Chi F. Shen S. H. Cheng H. P. Jing Y. X. Yanni Y. G. Dazzo F. B. (2005). Ascending migration of endophytic rhizobia, from roots to leaves, inside rice plants and assessment of benefits to rice growth physiology. *Appl. Environ. Microbiol.* 71 7271–7278. 10.1128/AEM.71.11.7271-7278.2005 16269768 PMC1287620

[B8] Christian N. Herre E. A. Mejia L. C. Clay K. (2017). Exposure to the leaf litter microbiome of healthy adults protects seedlings from pathogen damage. *Proc. R. Soc. B* 284:20170641. 10.1098/rspb.2017.0641 28679727 PMC5524495

[B9] Crocker E. V. Karp M. A. Nelson E. B. (2015). Virulence of oomycete pathogens from *Phragmites australis*-invaded and noninvaded soils to seedlings of wetland plant species. *Ecol. Evol.* 5 2127–2139. 10.1002/ece3.1468 26078850 PMC4461415

[B10] Emi M. Kenji F. (2013). A comparison of fungal endophytic community diversity in tree leaves of rural and urban temperate forests of Kanto district, eastern Japan. *Fungal Biol.* 117 191–201. 10.1016/j.funbio.2013.01.007 23537876

[B11] Eppinga M. B. Rietkerk M. Dekker S. C. Ruiter P. C. D. (2006). Accumulation of local pathogens: a new hypothesis to explain exotic plant invasions. *Oikos* 114 168–176. 10.1111/j.2006.0030-1299.14625.x

[B12] Fischer M. S. Rodriguez R. J. (2013). Fungal endophytes of invasive *Phagramites australis* populations vary in species composition and fungicide susceptibility. *Symbiosis* 61 55–62. 10.1007/s13199-013-0261-z

[B13] Flory S. L. Clay K. Thrall P. (2013). Pathogen accumulation and long-term dynamics of plant invasions. *J. Ecol.* 101 607–613. 10.1111/1365-2745.12078

[B14] Gazis R. Rehner S. Chaverri P. (2011). Species delimitation in fungal endophyte diversity studies and its implications in ecological and biogeographic inferences. *Mol. Ecol.* 20 3001–3013. 10.1111/j.1365-294X.2011.05110.x 21557783

[B15] Geisen S. Kostenko O. Cnossen M. C. Ten Hooven F. C. Vres B. van der Putten W. H. (2017). Seed and root endophytic fungi in a range expanding and a related plant species. *Front. Microbiol.* 8:1645. 10.3389/fmicb.2017.01645 28900420 PMC5581836

[B16] Glynou K. Ali T. Buch A. K. Haghi Kia S. Ploch S. Xia X. (2016). The local environment determines the assembly of root endophytic fungi at a continental scale. *Environ. Microbiol.* 18 2418–2434. 10.1111/1462-2920.13112 26530450

[B17] Grayston S. J. Wang S. Campbell C. D. Edwards A. C. (1998). Selective influence of plant species on microbial diversity in the rhizosphere. *Soil Biol. Biochem.* 30 369–378. 10.1016/s0038-0717(97)00124-7

[B18] Harman G. E. (2006). Overview of mechanisms and uses of *Trichoderma* spp. *Phytopathology* 96 190–194. 10.1094/PHYTO-96-0190 18943924

[B19] Hodgson S. de Cates C. Hodgson J. Morley N. J. Sutton B. C. Gange A. C. (2014). Vertical transmission of fungal endophytes is widespread in forbs. *Ecol. Evol.* 4 1199–1208. 10.1002/ece3.953 24834319 PMC4020682

[B20] Inderjit S. Evans H. Crocoll C. Bajpai D. Kaur R. Feng Y. L. (2011). Volatile chemicals from leaf litter are associated with invasiveness of a Neotropical weed in Asia. *Ecology* 92 316–324. 10.1890/10-0400.1 21618911

[B21] Jaber L. R. Vidal S. (2010). Fungal endophyte negative effects on herbivory are enhanced on intact plants and maintained in a subsequent generation. *Ecol. Entomol.* 35 25–36. 10.1111/j.1365-2311.2009.01152.x

[B22] Kazenel M. R. Kivlin S. N. Taylor D. L. Lynn J. S. Rudgers J. A. (2019). Altitudinal gradients fail to predict fungal symbiont responses to warming. *Ecology* 100:e02740. 10.1002/ecy.2740 31006112

[B23] Keane R. M. Crawley M. J. (2002). Exotic plant invasions and the enemy release hypothesis. *Trends Ecol. Evol.* 17 164–170. 10.1016/s0169-5347(02)02499-0

[B24] Kogel K. H. Franken P. Huckelhoven R. (2006). Endophyte or parasite - what decides? *Curr. Opin. Plant Biol.* 9 358–363. 10.1016/j.pbi.2006.05.001 16713330

[B25] Li Y. P. Feng Y. L. Kang Z. L. Zheng Y. L. Zhang J. L. Chen Y. J. (2017). Changes in soil microbial communities due to biological invasions can reduce allelopathic effects. *J. Appl. Ecol.* 54 1281–1290. 10.1111/1365-2664.12878

[B26] Lodge D. J. Fisher P. J. Sutton B. C. (1996). Endophytic fungi of *Manilkara bidentata* leaves in Puerto Rico. *Mycologia* 88 733–738. 10.2307/3760967

[B27] Lymperopoulou D. S. Adams R. I. Lindow S. E. Löffler F. E. (2016). Contribution of vegetation to the microbial composition of nearby outdoor air. *Appl. Environ. Microbiol.* 82 3822–3833. 10.1128/aem.00610-6 27107117 PMC4907200

[B28] Malinich E. Lynn-Bell N. Kourtev P. S. (2017). The effect of the invasive *Elaeagnus umbellata* on soil microbial communities depends on proximity of soils to plants. *Ecosphere* 8:e01827 10.1002/ecs2.1827

[B29] Martí R. E. Cubero J. Daza A. Piquer J. Salcedo C. I Morente C. (1999). Evidence of migration and endophytic presence of *Agrobacterium tumefaciens* in rose plants. *Eur. J. Plant pathol.* 105 39–50.

[B30] Martiny J. B. Bohannan B. J. Brown J. H. Colwell R. K. Fuhrman J. A. Green J. L. (2006). Microbial biogeography: putting microorganisms on the map. *Nat. Rev. Microbiol.* 4 102–112. 10.1038/nrmicro1341 16415926

[B31] Mei L. Zhu M. Zhang D. Z. Wang Y. Z. Guo J. Zhang H. B. (2014). Geographical and temporal changes of foliar fungal endophytes associated with the invasive plant *Ageratina adenophora*. *Microb. Ecol.* 67 402–409. 10.1007/s00248-013-0319-8 24276537

[B32] Mitchell C. E. Blumenthal D. Jarosik V. Puckett E. E. Pysek P. (2010). Controls on pathogen species richness in plants’ introduced and native ranges: roles of residence time, range size and host traits. *Ecol. Lett.* 13 1525–1535. 10.1111/j.1461-0248.2010.01543.x 20973907 PMC3003901

[B33] Mitchell C. E. Power A. G. (2003). Release of invasive plants from fungal and viral pathogens. *Nature* 421 625–627. 10.1038/nature01317 12571594

[B34] Mucciarelli M. Scannerini S. Bertea C. Maffei M. (2003). In vitro and in vivo peppermint (*Mentha piperita*) growth promotion by nonmycorrhizal fungal colonization. *New Phytol.* 158 579–591. 10.1046/j.1469-8137.2003.00762.x 36056516

[B35] Müller C. Krauss J. (2005). Symbiosis between grasses and asexual endophytes. *Curr. Opin. Plant Biol.* 8 450–456. 10.1016/j.pbi.2005.05.007 15946893

[B36] Newcombe G. Shipunov A. Eigenbrode S. Raghavendra A. Ding H. Anderson C. (2009). Endophytes influence protection and growth of an invasive plant. *Commun. Integr. Biol.* 2 29–31. 10.4161/cib.2.1.7393 19704862 PMC2649296

[B37] Newsham K. K. (2011). A meta-analysis of plant responses to dark septate root endophytes. *New Phytol.* 190 783–793. 10.1111/j.1469-8137.2010.03611.x 21244432

[B38] O’Connell R. J. Thon M. R. Hacquard S. Amyotte S. G. Kleemann J. Torres M. F. (2012). Lifestyle transitions in plant pathogenic *Colletotrichum* fungi deciphered by genome and transcriptome analyses. *Nat. Genet.* 44 1060–1065. 10.1038/ng.2372 22885923 PMC9754331

[B39] Photita W. Lumyong S. Lumyong P. McKenzie E. H. C. Hyde K. D. (2004). Are some endophytes of *Musa acuminata* latent pathogens? *Fungal Divers.* 16 131–140.

[B40] Ritpitakphong U. Falquet L. Vimoltust A. Berger A. Metraux J. P. L’Haridon F. (2016). The microbiome of the leaf surface of *Arabidopsis* protects against a fungal pathogen. *New Phytol.* 210 1033–1043. 10.1111/nph.13808 26725246

[B41] Rodriguez R. J. Henson J. Van Volkenburgh E. Hoy M. Wright L. Beckwith F. (2008). Stress tolerance in plants via habitat-adapted symbiosis. *ISME J.* 2 404–416. 10.1038/ismej.2007.106 18256707

[B42] Rodriguez R. J. White J. F. Jr. Arnold A. E. Redman R. S. (2009). Fungal endophytes: diversity and functional roles. *New Phytol.* 182 314–330. 10.1111/j.1469-8137.2009.02773.x 19236579

[B43] Romero A. Carrion G. Rico-Gray V. (2001). Fungal latent pathogens and endophytes from leaves of *Parthenium hysterophorus* (Asteraceae). *Fungal Divers.* 7 81–87.

[B44] Rudgers J. A. Mattingly W. B. Koslow J. M. (2005). Mutualistic fungus promotes plant invasion into diverse communities. *Oecologia* 144 463–671. 10.1007/s00442-005-0039-y 15942761

[B45] Ryberg M. (2015). Molecular operational taxonomic units as approximations of species in the light of evolutionary models and empirical data from Fungi. *Mol. Ecol.* 24 5770–5777. 10.1111/mec.13444 26523754

[B46] Saleem M. Meckes N. Pervaiz Z. H. Traw M. B. (2017). Microbial interactions in the phyllosphere increase plant performance under herbivore biotic stress. *Front. Microbiol.* 8:41. 10.3389/fmicb.2017.00041 28163703 PMC5247453

[B47] Sarmiento C. Zalamea P. C. Dalling J. W. Davis A. S. Stump S. M. U’Ren J. M. (2017). Soilborne fungi have host affinity and host-specific effects on seed germination and survival in a lowland tropical forest. *Proc. Natl. Acad. Sci. U.S.A.* 114 11458–11463. 10.1073/pnas.1706324114 28973927 PMC5664508

[B48] Schloss P. D. Westcott S. L. Ryabin T. Hall J. R. Hartmann M. Hollister E. B. (2009). Introducing mothur: open-source, platform-independent, community-supported software for describing and comparing microbial communities. *Appl. Environ. Microbiol.* 75 7537–7541. 10.1128/aem.01541-9 19801464 PMC2786419

[B49] Shi Y. Li C. Yang H. Zhang T. Gao Y. Zeng J. (2016). Endophytic fungal diversity and space-time dynamics in sugar beet. *Eur. J. Soil Biol.* 77 77–85. 10.1016/j.ejsobi.2016.09.005 24752839

[B50] Shipunov A. Newcombe G. Raghavendra A. K. Anderson C. L. (2008). Hidden diversity of endophytic fungi in an invasive plant. *Am. J. Bot.* 95 1096–1108. 10.3732/ajb.0800024 21632429

[B51] Stewart C. N. Jr. Via L. E. (1993). A rapid CTAB DNA isolation technique useful for RAPD fingerprinting and other PCR applications. *Biotechniques* 14 748–750. 8512694

[B52] Stricker K. B. Harmon P. F. Goss E. M. Clay K. Luke Flory S. (2016). Emergence and accumulation of novel pathogens suppress an invasive species. *Ecol. Lett.* 19 469–477. 10.1111/ele.12583 26931647

[B53] Ter Braak C. J. F. Šmilauer P. (2012). *Canoco Reference Manual and User’s Guide: Software for Ordination (version 5.0).* Wageningen: Microcomputer power.

[B54] Tharek M. Dzulaikha K. Salwani S. Amir H. G. Najimudin N. (2011). Ascending endophytic migration of locally isolated diazotroph, *Enterobacter* sp. strain USML2 in rice. *Biotechnology* 10 521–527. 10.3923/biotech.2011.521.527

[B55] Thompson J. D. Gibson T. J. Plewniak F. Jeanmougin F. Higgins D. G. (1997). The CLUSTAL_X windows interface: flexible strategies for multiple sequence alignment aided by quality analysis tools. *Nucleic Acids Res.* 25 4876–4882. 10.1093/nar/25.24.4876 9396791 PMC147148

[B56] Torres-Cortes G. Bonneau S. Bouchez O. Genthon C. Briand M. Jacques M. A. (2018). Functional microbial features driving community assembly during seed germination and emergence. *Front. Plant Sci.* 9:902. 10.3389/fpls.2018.00902 30008730 PMC6034153

[B57] van der Heijden M. G. Bardgett R. D. van Straalen N. M. (2008). The unseen majority: soil microbes as drivers of plant diversity and productivity in terrestrial ecosystems. *Ecol. Lett.* 11 296–310. 10.1111/j.1461-0248.2007.01139.x 18047587

[B58] van der Putten W. H. Bardgett R. D. Bever J. D. Bezemer T. M. Casper B. B. Fukami T. (2013). Plant-soil feedbacks: the past, the present and future challenges. *J. Ecol.* 101 265–276. 10.1111/1365-2745.12054

[B59] van der Putten W. H. Klironomos J. N. Wardle D. A. (2007). Microbial ecology of biological invasions. *ISME J.* 1 28–37. 10.1038/ismej.2007.9 18043611

[B60] Vilà M. Basnou C. Pyšek P. Josefsson M. Genovesi P. Gollasch S. (2010). How well do we understand the impacts of alien species on ecosystem services? A pan-European, cross-taxa assessment. *Front. Ecol. Environ.* 8:135–144. 10.1890/080083

[B61] Wang R. Wang Y.-Z. (2006). Invasion dynamics and potential spread of the invasive alien plant species *Ageratina adenophora* (Asteraceae) in China. *Divers. Distrib.* 12 397–408. 10.1111/j.1366-9516.2006.00250.x

[B62] Wang W. B. Wang R. F. Lei Y. B. Liu C. Han L. H. Shi X. D. (2013). High resource capture and use efficiency and prolonged growth season contribute to invasiveness of *Eupatorium adenophorum*. *Plant Ecol.* 214 857–868. 10.1007/s11258-013-0214-x

[B63] Wearn J. A. Sutton B. C. Morley N. J. Gange A. C. (2012). Species and organ specificity of fungal endophytes in herbaceous grassland plants. *J. Ecol.* 100 1085–1092. 10.1111/j.1365-2745.2012.01997.x

[B64] Whitaker B. K. Bauer J. T. Bever J. D. Clay K. (2017). Negative plant-phyllosphere feedbacks in native Asteraceae hosts - a novel extension of the plant-soil feedback framework. *Ecol. Lett.* 20 1064–1073. 10.1111/ele.12805 28677329

[B65] Xu C. W. Yang M. Z. Chen Y. J. Chen L. M. Zhang D. Z. Mei L. (2012). Changes in non-symbiotic nitrogen-fixing bacteria inhabiting rhizosphere soils of an invasive plant *Ageratina adenophora*. *Appl. Soil Ecol.* 54 32–38. 10.1016/j.apsoil.2011.10.021

[B66] Yahdjian L. Gherardi L. Sala O. E. (2011). Nitrogen limitation in arid-subhumid ecosystems: a meta-analysis of fertilization studies. *J. Arid Environ.* 75 675–680. 10.1016/j.jaridenv.2011.03.003

[B67] Yamamoto N. Bibby K. (2014). Clustering of fungal community internal transcribed spacer (ITS) sequence data obscures taxonomic diversity. *Environ. Microb.* 16 2491–2500. 10.1111/1462-2920.12390 24428124

[B68] Yan J. F. Broughton S. J. Yang S. L. Gange A. C. (2015). Do endophytic fungi grow through their hosts systemically? *Fungal Ecol.* 13 53–59. 10.1016/j.funeco.2014.07.005

[B69] Yokoya K. Postel S. Fang R. Sarasan V. (2017). Endophytic fungal diversity of *Fragaria vesca*, a crop wild relative of strawberry, along environmental gradients within a small geographical area. *PeerJ* 5:e2860. 10.7717/peerj.2860 28168102 PMC5289447

[B70] Zhou J. Miao Y. F. Fang K. Chen L. Yang Z. P. Dong X. F. (2019). Diversity of the endophytic and rhizosphere soil fungi of *Ageratina adenophora*. *Ecol. Sci.* 38 1–7. 10.14108/j.cnki.1008-8873.2019.05.001

[B71] Zhu X. Z. Li Y. P. Feng Y. L. Ma K. P. (2017). Response of soil bacterial communities to secondary compounds released from *Eupatorium adenophorum*. *Biol. Invasions* 19 1471–1481. 10.1007/s10530-017-1371-y

