# Supplementary material for: Tissue-Specific and Geographical Variation in Endophytic Fungi of Ageratina adenophora and Fungal Associations With the Environment
Source: Front Microbiol. 2019 Dec 18;10:2919. doi: 10.3389/fmicb.2019.02919 (PMC6930192; doi:10.3389/fmicb.2019.02919)
Supplement: Supplementary file 1 [file Image_1.pdf]

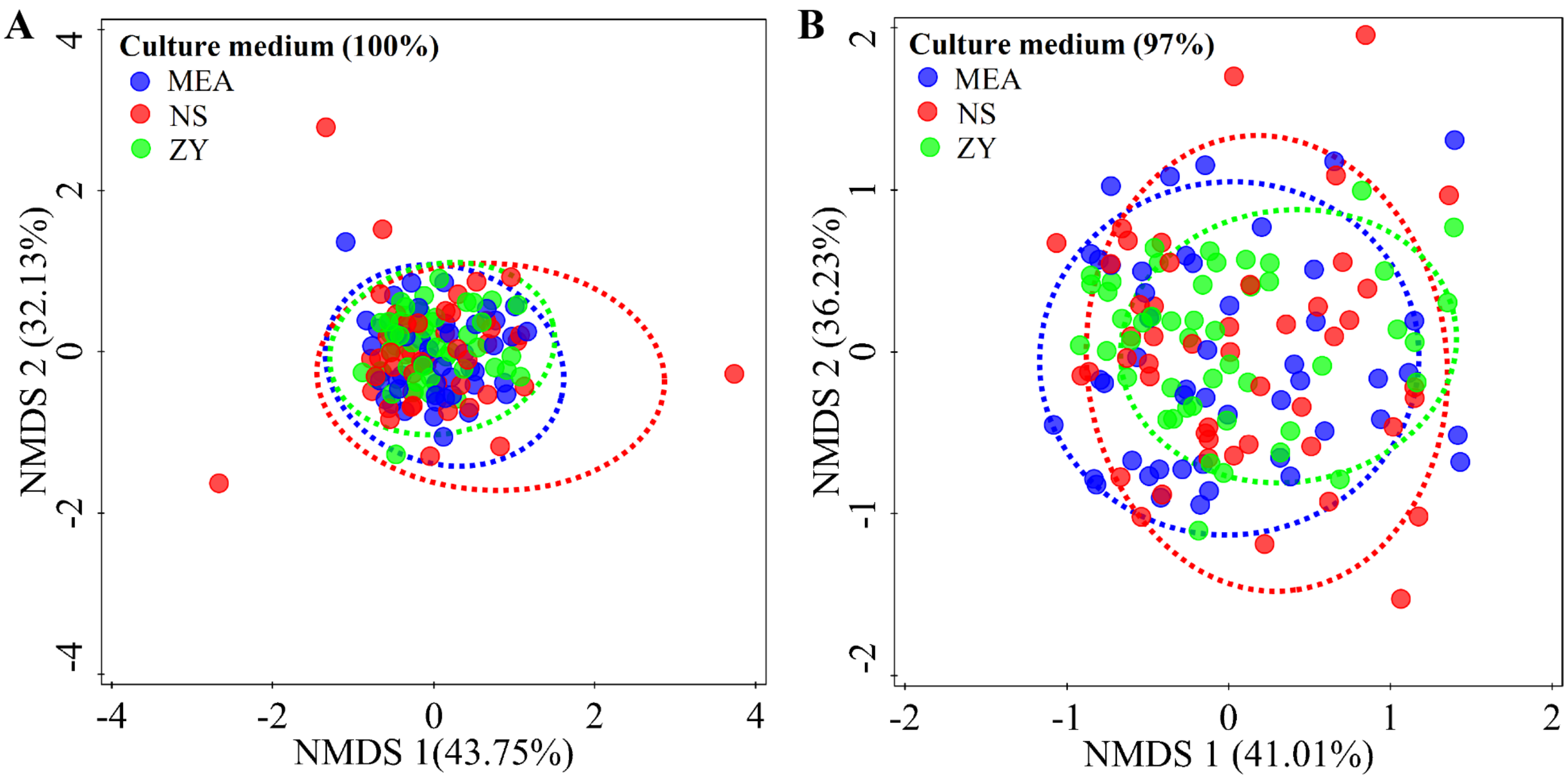

Supplementary Figure S1. Fungal communities isolated from different culture media. Nonmetric multidimensional scaling (NMDS) analysis shows the similarity among the cultivable fungal communities (points) isolated from all samples, including plant tissue, air and soil. The points with different colors represent different culture media. Panel A (stress = 0.064) and B (stress = 0.109) show the results at the unique and 97% sequence identity levels, respectively. In order to visualize the primary data better, the NMDS analysis shown in panel A does not take two outliers into account, including NE-CA-ZY and NE-AS-MEA (NE represents sampling site, CA (canopy air) and AS (ambient soil) represent isolation source, ZY and MEA represent culture medium). Percentages of total variation explained by NMDS axes are given in parentheses. Redundancy analyses show that medium types are not significant when sequence analyses are performed at the unique level ($F = 0.9$; $p = 0.624$; 0.7% explained variation, adjusted explained variation 0.0%), but significant at 97% sequence identity level ($F = 2.2$; $p = 0.002$; 1.5% explained variation, adjusted explained variation 0.8%).
