# Supplementary material for: Tissue-Specific and Geographical Variation in Endophytic Fungi of Ageratina adenophora and Fungal Associations With the Environment
Source: Front Microbiol. 2019 Dec 18;10:2919. doi: 10.3389/fmicb.2019.02919 (PMC6930192; doi:10.3389/fmicb.2019.02919)
Supplement: Supplementary file 2 [file Image_2.pdf]

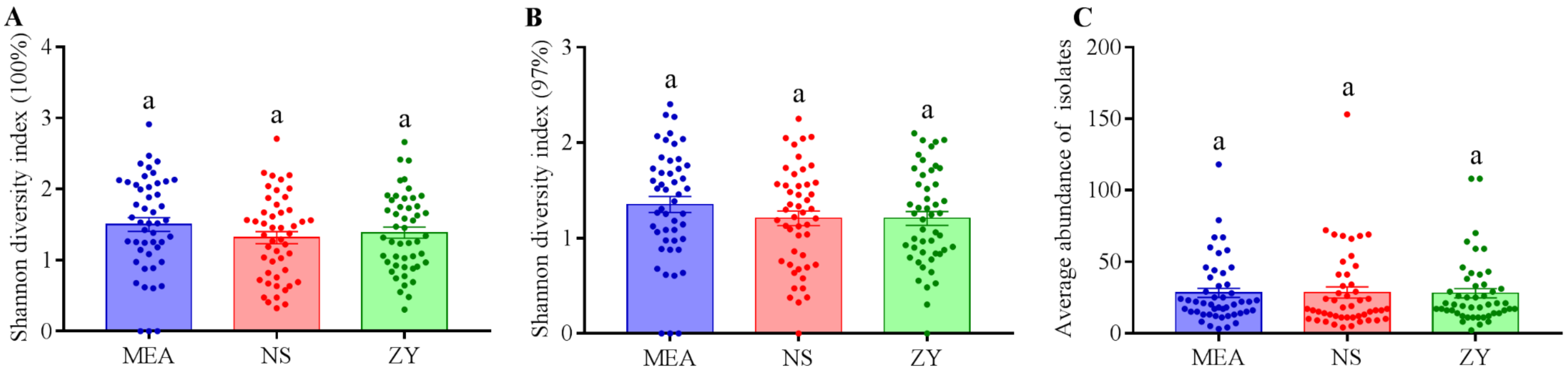

Supplementary Figure S2. Variation in Shannon diversity index (A (unique), B (97%)) and average abundance (C) for the cultivable fungal communities using different media. To satisfy normality and homoscedasticity assumptions, a log-transformation was applied to average abundance. Duncan was used for multiple comparisons. The same lowercase letters indicated no significant difference (p >0.05). Error bars depict the standard error.
