# Supplementary material for: Tissue-Specific and Geographical Variation in Endophytic Fungi of Ageratina adenophora and Fungal Associations With the Environment
Source: Front Microbiol. 2019 Dec 18;10:2919. doi: 10.3389/fmicb.2019.02919 (PMC6930192; doi:10.3389/fmicb.2019.02919)
Supplement: Supplementary file 3 [file Image_3.pdf]

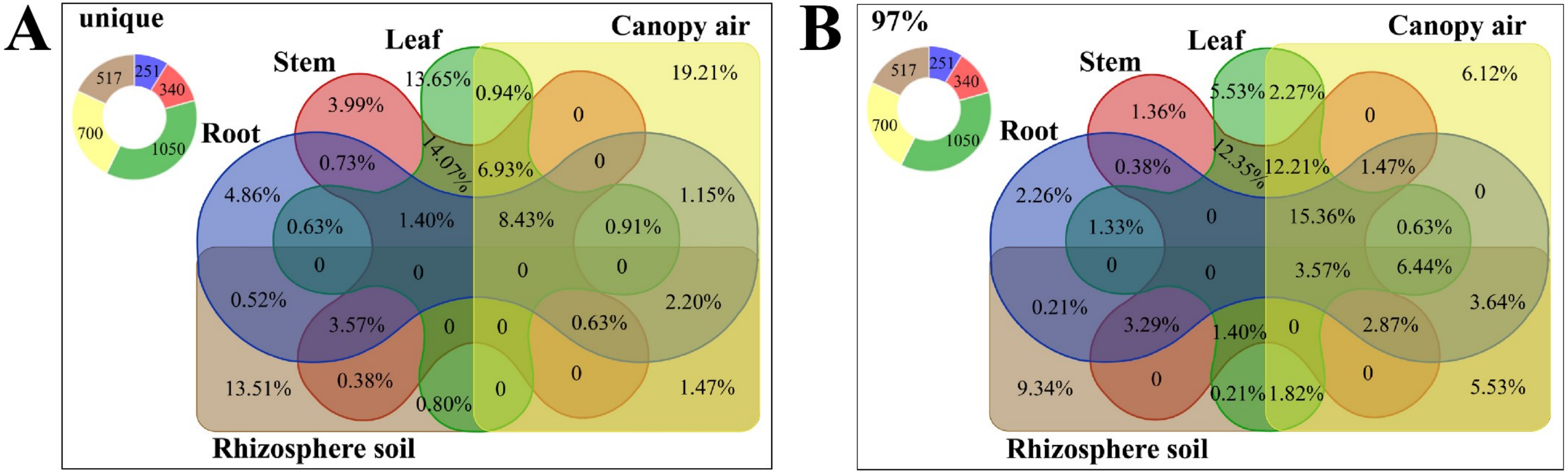

Supplementary Figure S3. Relative abundance of cultivable fungi among plant tissue, canopy air and rhizosphere soil at the unique (A) and 97% (B) sequence identity levels. The overlapping parts of solid shapes with different colors represents the same OTUs, and the percentage is the relative abundance of fungi. The different color parts of ring diagrams show the abundance of cultivable endophytic fungi (including root, stem and mature leaf), rhizosphere fungi and canopy air fungi, respectively.
